# The gait pattern is not impaired in subjects with external snapping hip: a comparative cross-sectional study

**DOI:** 10.1186/1471-2474-14-212

**Published:** 2013-07-19

**Authors:** Julie S Jacobsen, Uwe G Kersting, Michael S Rathleff, Ole Simonsen, Kjeld Søballe, Michael Ulrich

**Affiliations:** 1 Department of Physiotherapy and Occupational Therapy, Aarhus University Hospital, Tage-Hansens Gade 2, 8000, Aarhus C, Denmark; 2 HammelNeurorehabilitation Hospital and Research Center, Aarhus University, Voldbyvej 15, 8450, Hammel, Denmark; 3 Center for Sensory-Motor Interaction, Department of Health Science and Technology, Aalborg University, Fredrik Bajers Vej 7 D3, 9220, Aalborg, Denmark; 4 Orthopaedic Surgery Research Unit, Aalborg University Hospital, Hobrovej 18-22, 9100, Aalborg, Denmark; 5 Department of Orthopaedic Surgery, Aalborg University Hospital, Hobrovej 18-22, 9100, Aalborg, Denmark; 6 Department of Orthopaedic Research, Aarhus University Hospital, Tage-Hansens Gade 2, 8000, Aarhus C, Denmark

**Keywords:** Iliotibial band, Trochantor pain, Healthy subjects, Walking, Electromyography, EMG

## Abstract

**Background:**

Symptomatic external snapping hip is a painful condition, where pain in the trochantor region and limitations of daily activity dominate clinical findings. The aetiology of symptomatic external snapping hip is elusive, but previous studies have suggested that weakness of the hip abductors and an altered walking pattern may play a role in the development of symptomatic external snapping hip. The aim of this study was to compare the walking pattern and muscular activity of the hip muscles between subjects with symptomatic external snapping hip and healthy subjects.

**Methods:**

Thirteen subjects with diagnosed symptomatic external snapping hip (age: 25.5 years) were matched with 13 healthy subjects (age: 25.6 years). Joint kinematics and kinetics of the lower extremity were quantified by the peak hip adduction angle; the average knee rotation range of motion (ROM) and the peak valgus knee angle after data recording using a Vicon 612 motion capture system. Muscle activity was recorded bilaterally using surface electromyography (sEMG) on five muscles: gluteus maximus, gluteus medius, tensor fascia latae, rectus femoris and biceps femoris. A paired *t*-test was used to evaluate differences between the two groups.

**Results:**

No significant differences were found between the groups concerning the peak hip adduction angle, the average knee rotation ROM, and the static valgus knee angle. No significant between-group differences were found concerning all other kinematics, kinetics or muscle activity. In subjects with symptomatic external snapping hip activity of the gluteus medius muscle during the acceptance phase of walking was 0.58 ± 0.19 whereas the activity was 0.68±0.07 in the asymptomatic group (p=0.115).

**Conclusions:**

No significant differences in the walking pattern were found between subjects with symptomatic external snapping hip and healthy subjects. This suggest that subjects with symptomatic external snapping hip does not have an impaired gait pattern.

## Background

External snapping hip is present when the iliotibial tract and/or the posterior border of gluteus maximus slide over the greater trochanter [1-3]. Symptomatic external snapping hip is a painful condition, where pain in the trochantor region and limitations of daily activity dominate clinical findings [1,4,5]. Despite this the literature on the topic is surprisingly sparse.

The aetiology of external snapping hip is elusive, but previous studies have suggested that weakness of the hip abductors, leg length, tightness of the iliotibial band, foot mechanics, altered walking and hip instability may play a role in the development of the condition [3,6-9]. During walking the hip abductors work eccentrically after initial foot contact to resist an external adduction moment [10,11]. If the joint’s range of motion is increased as in generalised joint hypermobility these muscles work even harder, and this can result in musculoskeletal pain problems [12]. Previous studies have described deficits of the gluteal muscles being related to altered kinematics and kinetics during walking [10,13]; but the association has, to our knowledge, never been documented with valid outcome measures in subjects with symptomatic external snapping hip.

External snapping hip involves the iliotibial tract, with the primary role of the tract being to resist hip adduction and knee rotation during walking and running [14]. A consequence of the proximal and distal attachments of the tract may be that external snapping is associated with increased hip adduction and increased knee rotation as it has been documented in runners with iliotibial band syndrome [15]. In addition, the activity of the gluteus medius muscle may be affected due to pain in the trochantor region as it has been found in healthy people with induced experimental pain [16,17].

To our knowledge, only few published studies report the clinical symptoms of external snapping hip. One study on external snapping hip reported an increased hip adduction angle and increased valgus knee angle during walking [7]. Furthermore, bilateral subtalar over-pronation accompanied by an increased static valgus knee angle in a standing position was found in the same study. Another study by Jacobsen et al. [9] reported decreased eccentric hip abduction strength in subjects with external snapping hip, and Bellabarba et al. [6] reported a high prevalence of hip instability in subjects with internal snapping hip. These studies indicate important impairments related to snapping hip, but the case report by Spine [7] fails to prove an association between walking and external snapping hip because of its design and the use of non-valid outcome measures.

The aim of this study was to compare the walking pattern and muscular activity of the hip muscles between subjects with symptomatic external snapping hip and healthy subjects. We hypothesised, that subjects with symptomatic external snapping hip would present with an increased hip adduction angle, an increased average knee rotation Range of Motion (ROM), an increased valgus knee angle and reduced activity of the gluteus medius muscle during the stance phase of walking.

## Methods

### Subjects

The number of subjects was based on a priori sample size calculation based on a previous study evaluating kinematics and kinetics in runners with lateral hip pain [15]. Based on a mean value of the peak hip adduction angle of 14.1 (SD 2.5) degrees in subjects with external snapping hip and 10.6 (SD 3.6) degrees in healthy subjects it was found that a minimum of 13 subjects in each group were needed. The sample size calculation was based on a significance level of 5% and a power of 80%.

Thirteen subjects with external snapping hip (six men and seven women) were recruited from the Division of Hip Surgery at the Department of Orthopaedics at Aarhus University Hospital, Denmark between 1 April 2010 to 1 November 2010 (Table 1). The subjects had a diagnosis of unilateral or bilateral symptomatic external snapping hip, and had to meet the following criteria: an external painful snap from the lateral hip region within the last fourteen days, a clinical diagnosis of external snapping hip [1], self-reported pain at the greater trochantor or in the gluteal region marked on a self-reported pain drawing at the time of inclusion [18], between 18 and 50 years of age. The exclusion criteria were: osteoarthritis in the hip joint, hip dysplasia, internal snapping hip, other intra-articular pathology, previous operations in the hip, knee or ankle regions, diseases or injuries affecting muscle strength in the legs, pregnancy, steroid injection in the hip region within the last month. The diagnosis of external snapping hip was given, when a palpable snap was felt in the trochantor region, and if the snap was reported as painful by the subject during a side-lying extension and flexion movement. The diagnosis was also given, when the painful snap was reproduced in a standing position. The subjects were diagnosed by one of two experienced hip orthopedic surgeons and afterwards the inclusion and exclusion criteria were evaluated by JSJ on all subjects. Parallel to the inclusion of subjects with external snapping hip, a control group of 13 subjects (six men and seven women) with no hip, knee, ankle or back problems were included from the social network of the subjects with external snapping hip and through the hospital’s intranet. The two groups were matched based on gender and age (± five years). All subjects consented to participate in the study, and the Central Denmark Region Committees on Biomedical Research Ethics (M-20090094) and the Danish Data Protection Agency (2007-58-0010) approved the study.

**Table 1:** Baseline characteristics

	**SESH n = 13**	**Controls n = 13**	**P-value**
Bilateral/unilateral n	7/6	-	-
Duration of pain years (IQR)	2.5 (1.5-5.0)	-	-
Age years	25.5 ± 3.4	25.6 ± 2.6	-
Rest 100 mm VAS (IQR)	4 (0–10)	0 (0–0)	0.010
Activity 100 mm VAS	10 (0–20)	0 (0–0)	0.010
Height meters	1.8 ± 0.1	1.7 ± 0.1	0.291
Leg length meters	0.89 ± 0.05	0.91 ± 0.05	0.221
Weight kilo	70.9 ± 17.2	68.4 ± 9.8	0.571
BMI kilo/meters^2^	23.1 ± 4.2	23.0 ± 3.0	0.946
Non-prescriptive medication n	6 (46.2%)	0 (0.0%)	0.031
Prescriptive medication n	0 (0.0%)	0 (0.0%)	1.000
Leg dominance, right n	12 (92.3%)	9 (69.2%)	0.250
Positive FPI (pronation) n	8 (61.5%)	5 (38.5%)	0.453

Baseline characteristics in subjects with external snapping hip (SESH) and in healthy subjects (controls) are presented as median (IQR), as mean ± SD (Standard Deviation) and as number and prevalence (%).

*Abbreviations*: *IQR* (Interquartile Range), *100 mm VAS* (100 millimeter Visual Analog pain Scale), *BMI* (Body Mass Index), *FPI* (Foot Posture Index).

### Design and procedure

This study is a comparative cross-sectional study of walking in subjects with external snapping hip and healthy subjects. This study is one out of two parallel cross-sectional studies on the same subjects. The two parallel studies were designed simultaneously but with individual outcome measures and hypotheses. One evaluated hip muscle strength and patient-reported outcomes using a dynamometer and the questionnaire Hip dysfunction and Osteoarthritis Outcome Score (HOOS) [9]. This study evaluated specific mechanical outcomes related to walking.

In the present study the dynamic activity of the hip muscles, joint kinematics and kinetics of the lower extremity were compared between subjects with external snapping hip and healthy subjects. The primary outcomes were the hip adduction angle, the average knee rotation ROM and the activity of the gluteus medius muscle. Additionally, the valgus knee angle was determined from a static trial.

Baseline characteristics were registered using standardized questions. Weight was measured on a calibrated scale, and height was measured on a wall-mounted ruler. Leg length was measured by an experienced physiotherapist as the distance from the trochantor major to the lateral malleolus on both sides. Limp dominance was reported by asking the subjects which limp they would use if they were going to kick a ball. Prior to testing, pain was rated during rest by the subjects on a 100 mm Visual Analogue pain Scale (VAS) [19]. Likewise, subjects were asked about pain during the test trials. Furthermore, ankle posture was examined using The Foot Posture Index 6 (FPI-6) [20]. In addition to the baseline characteristics, general joint hypermobility was examined as a secondary outcome measure using Beighton’s test of general joint hypermobility [21]. A subject had general joint hypermobility if five out of nine criteria were fulfilled [21].

### Three-dimensional gait analysis

Walking was recorded with the Vicon 612 motion capture system using eight cameras (Vicon Motion System Limited, Oxford, UK). Optic data were sampled at 100 Hz, and the Vicon plug-in gait marker protocol was used totalling thirty-nine 8-mm polypropylene retroreflective markers. Three extra markers were attached to bone prominences corresponding to the calcaneus and the head of the first and fifth metatarsal. Ground reaction forces were sampled at 2000 Hz using an OR6-7 AMTI force plate (AMTI, Advanced Mechanical Technology, Watertown, USA).

Activity of the hip muscles was recorded with surface electromyography (sEMG) from the gluteus maximus, the gluteus medius, the tensor fascia latae, the rectus femoris and the biceps femoris. Skin preparation and electrode placement was conducted according to the SENIAM recommendations [22] in a bipolar derivation with Ag/AgCl electrodes (AmbuNeuroline 720 01-K/12; Ambu, Ballerup, Denmark) with 22 mm of centre-to-centre spacing. Prior to electrode placement the skin was shaved and cleansed with alcohol. The sEMG data were recorded simultaneously with the motion capture analysis using a MA300 Advanced Multi-Channel EMG System (Motion Lab Systems, Inc., Los Angeles, USA). The sEMG data were sampled at 2000 Hz and collected synchronously with ground reaction forces.

The subjects walked barefoot at a self-selected speed along a 10-m walkway. The camera system was calibrated before each testing day producing residual errors less than one millimeter over a volume of 10 m × 3 m × 3 m. A static reference trial was recorded to adjust the marker set to individual anatomy and to evaluate valgus knee angle. Six right and six left dynamic trials were recorded, where the subject had to hit the force plate with the whole foot. Right or left trials were selected for statistical analysis based on the affected leg. In patients with bilateral involvement the trials of the worst affected leg, as reported by the subject, were selected. The corresponding trials (i.e., same leg) of the matched healthy subjects were selected for analysis. The force plate was covered with the same vinyl surface as the laboratory floor and not noticeable. Subjects were naive to gait analysis, and they were not informed about the requirement of hitting the force plate. The start position was altered, if necessary, and marked after the subjects had executed a sufficient number of trials to hit the plate (3 – 12 familiarization trials). Once the walk up distance was defined the subjects were vocally addressed to get ready and start walking for data recording. We only experienced a small number of trials where the subjects did not hit the force plate, and the maximum number of test trials was 16 for the whole sample. Experience from previous investigations in the laboratory demonstrated a good repeatability in participants with relatively small restrictions in gait function. Centre of mass velocity was determined during the analysis and varied by maximum +/− 3.7% in standard deviation.

### Data processing

Marker trajectories were filtered with a Woltring filter routine following Vicon’s recommendations. The smoothed marker coordinate data were analyzed using plug-in gait inverse dynamics model and results extracted using a MatLab script (version 2010b, The MathWorks Inc., Natick, MA). In the sagittal plane flexion were assigned as a positive value, in the frontal plane adduction was assigned as a positive value and in the horizontal plane internal rotation was assigned as a positive value. The gait cycle was time-normalized to 100%, and events were automatically estimated by the program and manually corrected if necessary. Average maximum and minimum angles and net joint moments during selected phases of the gait cycle and while standing were calculated for the pelvis, hip, knee and ankle.

The sEMG signals were band-pass filtered at 20–500 Hz. The raw sEMG signals were low-pass filtered with a cut-off limit of 10 Hz to create linear envelopes. The linear envelopes were normalized in amplitude to the maximum sEMG signal during walking found across all trials from one subject and expressed as percent-maximum amplitudes [23]. From the linear normalized curves mean amplitudes were calculated in the following time periods: sEMG-preactivation (150 ms), sEMG-acceptance, sEMG-mid stance, sEMG-late stance and sEMG-swing phase.

### Statistical analysis

Scatter-plots and histograms were used to confirm normal distribution of baseline characteristics and outcomes. Normally distributed baseline characteristics were presented as mean ± one standard deviation (SD). Otherwise baseline characteristics were presented as median and interquartile range (IQR). For the normally distributed data a paired *t*-test was used to evaluate differences between the groups, and otherwise non-parametric tests were used. The categorical baseline characteristics were presented as prevalence and prevalence proportion ratio (PPR) and the exact McNemar’s probability test was used to test differences between the groups. All outcomes are presented as mean ± SD and mean differences between the groups are presented with a 95% confidence interval (CI).

## Results

The subjects with external snapping hip reported higher VAS pain scores and use of pain medications compared to the healthy subjects (Table 1). In Beighton’s test of joint hypermobility six subjects with external snapping hip (46.2%) fulfilled the criteria of general joint hypermobility compared to one healthy subject (7.7%), the difference in prevalence was not significant (p = 0.063).

### Gait analysis

No differences were found between the groups concerning speed and step length (Table 2). Similary, no significant differences were found between the groups concerning the peak hip adduction angle and the average knee rotation ROM (Table 3). Furthermore, no difference was found regarding the static valgus knee angle, and no significant differences were found between groups concerning all other kinematic variables (Table 3). There were no significant differences for dynamic valgus knee angles (Table 3). The statistical analysis showed no significant differences between the two groups with regard to the kinetic data (Table 4).

**Table 2:** Spatio-temporal gait variables

	**SESH n = 13**	**Controls n = 13**	**Difference (95% CI)**	**P-value**
Gait speed m/s	1.3 ± 0.2	1.4 ± 0.1	0.1 (−0.0, 0.1)	0.159
Step length m	0.7 ± 0.1	0.7 ± 0.1	0.0 (−0.0, 0.1)	0.657

Spatio-temporal gait data are presented in the patients with external snapping hip (SESH) and healthy subjects (controls). The differences are reported as 95% CI (95% confidence interval).

**Table 3:** Kinematic gait variables

	**SESH n = 13**	**Controls n = 13**	**Difference (95% CI)**	**P-value**
Hip adduction angle^1^	4.5 ± 2.8	4.0 ± 2.8	−0.1 (−2.6, 1.6)	0.600
Hip abduction angle^2^	−2.7 ± 3.0	−3.0 ± 2.3	−0.2 (−2.5, 2.0)	0.821
Hip flexion angle ^1^	30.0 ± 5.8	31.8 ± 4.5	1.7 (−3.6, 7.0)	0.490
Hip extension angle^3^	−13.5 ± 6.8	−13.1 ± 6.9	0.4 (−5.1, 5.8)	0.892
Hip intern rotation angle^4^	9.4 ± 6.7	8.4 ± 8.2	−0.9 (−7.9, 6.1)	0.777
Hip extern rotation angle^5^	0.5 ± 6.9	0.2 ± 5.7	−0.3 (−7.1, 6.6)	0.937
Hip extern rotation angle^6^	1.7 ± 8.4	3.1 ± 6.4	1.4 (−5.9, 8.8)	0.684
Knee flexion angle^7^	14.8 ± 4.6	14.0 ± 4.8	−0.8 (−5.6, 3.9)	0.708
Knee rotation (ROM)^8^	12.0 ± 4.1	12.7 ± 4.1	0.7 (−3.5, 4.8)	0.731
Static knee valgus^9^	3.0 ± 2.6	3.7 ± 1.5	0.7 (−1.0, 2.4)	0.410
Dynamic knee valgus (ROM)^8^	6.4 ± 3.7	5.8 ± 3.2	−0.6 (−3.7, 2.5)	0.667
Dynamic knee valgus^2^	2.9 ± 3.0	3.6 ± 1.7	0.7 (−0.8, 2.3)	0.447
Plantar flexion angle^1^	12.8 ± 1.7	12.5 ± 2.8	−0.3 (−2.0, 1.4)	0.695
Dorsal flexion angle^5^	−5.8 ± 1.8	−6.2 ± 2.5	−0.4 (−2.1, 1.2)	0.568
Dorsal flexion angle^6^	−14.9 ± 5.8	−17.4 ± 3.9	−2.6 (−6.1, 1.0)	0.138
Foot progression external rotation^5^	−11.1 ± 9.3	−11.5 ± 4.4	−0.4 (−6.1, 5.3)	0.884
Pelvis lateral tilt^1^	5.6 ± 1.6	5.7 ± 2.4	0.2 (−1.4, 1.8)	0.834
Pelvis medial tilt^1^	−5.7 ± 2.2	−5.1 ± 2.0	0.7 (−1.0, 2.3)	0.414
Pelvis anterior tilt^1^	10.5 ± 4.5	11.7 ± 4.7	1.2 (2.9, 5.3)	0.529
Pelvis posterior tilt^1^	(+)7.7 ± 4.4	(+)9.5 ± 4.8	1.8 (−2.1, 5.8)	0.336
Pelvis internal rotation^1^	5.9 ± 2.5	4.9 ± 2.0	−1.1 (−3.1, 1.0)	0.280
Pelvis external rotation^1^	−4.1 ± 3.1	−2.9 ± 2.9	1.1 (−1.2, 3.4)	0.316

Kinematic data are presented in degrees as mean ± one standard deviation (SD). The difference between the patients with external snapping hip (SESH) and healthy subjects (controls) are reported as 95% CI (95% confidence interval).

1: Maximum during stance; 2: Touch down (0% stance); 3: Minimum during stance; 4: Maximum in 50% stance; 5: Minimum during 0-10% stance; 6: Minimum during 50-100% stance; 7: Maximum in 50% stance; 8: ROM (Range Of Motion) = Difference between maximum and minimum during stance; 9: Maximum in a static standing position (+) Positive: no posterior tilt occurs from the neutral position.

**Table 4:** Kinetic gait variables

	**SESH n = 13**	**Controls n = 13**	**Difference (95% CI)**	**P-value**
Hip adduction moment^1^	0.76 ± 0.14	0.76 ± 0.17	0.00 (−0.14, 0.15)	0.976
Hip abduction moment^2^	−0.34 ± 0.18	−0.34 ± 0.12	0.00 (−0.14, 0.15)	0.947
Hip flexion moment^1^	0.99 ± 0.19	1.13 ± 0.29	0.14 (−0.05, 0.33)	0.129
Hip extension moment^2^	−0.92 ± 0.26	−0.91 ± 0.17	0.02 (−0.17, 0.21)	0.838
Hip intern rotation moment^1^	0.16 ± 0.03	0.17 ± 0.05	0.01 (−0.02, 0.05)	0.384
Hip extern rotation moment^2^	−0.10 ± 0.05	−0.10 ± 0.03	0.00 (−0.04, 0.04)	0.942
Knee flexion moment^1^	0.59 ± 0.21	0.54 ± 0.19	−0.04 (−24, 0.15)	0.637
Knee extension moment^3^	−0.45 ± 0.13	−0.52 ± 0.14	−0.07 (−0.19, 0.05)	0.241
Knee extension moment^4^	−0.12 ± 0.12	−0.11 ± 0.10	0.01 (−0.10, 0.12)	0.838
Knee internal rotation moment^1^	0.17 ± 0.04	0.20 ± 0.05	0.02 (−0.01, 0.06)	0.097
Knee external rotation moment^2^	−0.01 ± 0.01	−0.02 ± 0.01	0.00 (−0.01, 0.01)	0.530
Knee varus moment^1^	0.47 ± 0.09	0.52 ± 0.11	0.05 (−0.02, 0.12)	0.172
Plantar flexion moment^1^	1.55 ± 0.17	1.62 ± 0.22	0.06 (−0.07, 0.19)	0.331
Dorsal flexion moment^2^	−0.16 ± 0.05	−0.18 ± 0.06	−0.03 (−0.07, 0.01)	0.175

Kinetic data are presented in N*m/kg ± one standard deviation (SD). The difference between the patients with external snapping hip (SESH) and healthy subjects (controls) are reported as 95% CI (95% confidence interval).

1: Maximum during stance; 2: Minimum during stance; 3: Minimum during 0-10% stance; 4: Minimum during 50-100% stance.

### Electromyography

The sEMG data showed no significant differences between the groups (Table 5). In subjects with symptomatic external snapping hip activity of the gluteus medius muscle during the acceptance phase of walking was 0.58 ± 0.19 whereas the activity was 0.68±0.07 in the asymptomatic group (p=0.115).

**Table 5:** Electromyography (sEMG) gait variables

	**Preactivation**		**Acceptance**		**Middle stance**		**Late stance**		**Swing phase**	
	**Group mean**	**P-value**	**Group mean**	**P-value**	**Group mean**	**P-value**	**Group mean**	**P-value**	**Group mean**	**P-value**
Gluteus medius (S)	0.41 ± 0.23	0.312	0.58 ± 0.19	0.115	0.24 ± 0.11	0.818	0.16 ± 0.11	0.198	0.26 ± 0.14	0.262
Gluteus medius (H)	0.34 ± 0.12		0.68 ± 0.07		0.25 ± 0.10		0.11 ± 0.05		0.20 ± 0.09	
Gluteus maximus (S)	0.46 ± 0.18	0.593	0.62 ± 0.22	0.473	0.37 ± 0.17	0.288	0.34 ± 0.19	0.502	0.37 ± 0.13	0.433
Gluteus maximus (H)	0.49 ± 0.11		0.66 ± 0.12		0.32 ± 0.13		0.30 ± 0.12		0.33 ± 0.10	
Rectus femoris (S)	0.40 ± 0.17	0.092	0.64 ± 0.18	0.884	0.20 ± 0.07	0.819	0.20 ± 0.06	0.309	0.22 ± 0.05	0.204
Rectus femoris (H)	0.51 ± 0.19		0.65 ± 0.10		0.21 ± 0.06		0.25 ± 0.11		0.25 ± 0.08	
Biceps femoris (S)	0.51 ± 0.23	0.338	0.38 ± 0.18	0.494	0.20 ± 0.10	0.247	0.14 ± 0.13	0.209	0.33 ± 0.09	0.274
Biceps femoris (H)	0.59 ± 0.21		0.44 ± 0.16		0.15 ± 0.07		0.10 ± 0.04		0.30 ± 0.10	
Tensor Fascia latae (S)	0.32 ± 0.16	0.757	0.61 ± 0.15	0.154	0.34 ± 0.17	0.820	0.23 ± 0.16	0.674	0.33 ± 0.16	0.238
Tensor Fascia latae (H)	0.34 ± 0.12		0.68 ± 0.72		0.35 ± 0.12		0.21 ± 0.09		0.26 ± 0.09	

The sEMG data are presented as mean ± one standard deviation (SD) in 13 subjects with external snapping hip (S) and 13 healthy subjects (H) in the following time periods: preactivation (50 ms before heel strike), acceptance (load acceptance and initial single support), middle stance (knee extension and late single support), late stance (opposite foot contact) and swing phase (ipsilateral swing). The sEMG signals are normalized in amplitude to the maximum sEMG signal during gait and expressed as percent-maximum amplitudes.

## Discussion

The purpose of this study was to compare the walking pattern and muscular activity of the hip muscles during walking between subjects with symptomatic external snapping hip and healthy subjects. Comparing healthy hips with external snapping hips, we did not find neither an increased hip adduction angle nor an increased average knee rotation ROM during walking nor an increased static valgus knee angle in standing. However, we did observe a non-significant 10% reduction of the relative activity of the gluteus medius and the rectus femoris muscle among subjects with external snapping hip compared to the healthy subjects during stance. Some of the comparisons may be slightly underpowered but generally the differences between the groups were small, and therefore they may not be clinically relevant.

Spina [7] described a walking pattern with increased ipsilateral hip adduction and valgus knee angle together with lowering of the contralateral pelvis. The walking pattern was evaluated qualitatively by visual inspection in only one patient. It is possible that subjects with symptomatic external snapping hip modify walking differently, and this may explain the higher variability of the activity of the gluteus medius muscle among our subjects with external snapping hip compared to the healthy subjects. On the contrary, a detailed examination of the rectus femoris muscle revealed that an outlier among the healthy subjects was responsible for the difference between the groups indicating that the difference of the rectus femoris muscle is not consistent.

The trend towards a reduced sEMG activity of the gluteus medius muscle may be explained by a central inhibition mechanism caused by nociceptive inputs. Previous studies have found that experimental muscle pain is associated with a reduced activity of a muscle probably as a consequence of central inhibition of motor neurons [16,17,24]. Henriksen et al. [16] found that a pain avoidance pattern expressed as reduced muscle activity was present during, and after experimentally induced muscle pain which was confirmed in a later experimental study by Henriksen et al. [17]. These findings are further supported by Graven-Nielsen et al. [24] who demonstrated that the neural motor system might reorganize to protect a painful muscle by reducing the activity of the muscle. The question that should be raised, is if the tendency of reduced muscle activity is a result of pain or reduced hip abduction strength as it was reported by Jacobsen et al. [9] or a contributing cause of symptomatic external snapping hip? The current study design does not allow for any firm conclusion regarding the cause-effect relationship between pain, hip strength and muscle activity. Further studies on associations between external snapping hip and activity of the gluteus medius muscle during weight acceptance appear relevant.

Our results showed no significant differences between groups in the joint moments of the lower limb. Simonsen et al. [12] previously showed an association between joint moments and hypermobility indicating greater joint moments among subjects with hypermobility. Since almost half of our subjects with external snapping hip had general joint hypermobility it was expected, that the joint moments of the knee and hip in the frontal and sagittal plane would be increased. Surprisingly, this could not be confirmed, which from a patient point of view is positive as higher joint moments has been associated with pain in muscles and joints [16,25].

Forty-five percent of the subjects with external snapping were hypermobile [6]. Bellabarba et al. [6] reported hip instability defined as generalized ligamentous laxity in patients with pain and snapping in the groin tested with traction under fluoroscopy. They suggested that hip instability could play a role in the development of internal snapping hip as subclinical instability associated with ligamentous laxity may allow the iliopsoas to snap abnormally over structures just deep to it. In external snapping hip the iliotibial band snap over the trochantor major, which may cause an eccentric overload of the gluteals and the iliotibial band during movement as it was reported by Simonsen et al. [12]. However, other studies suggest that tightness of the iliotibial band is one of the main contributing factor to iliotibial snapping [3,5]. Further investigations are needed to explore a possible relationship between snapping hip and joint hypermobility.

### Clinical implications

Evidence of treating external snapping hip is sparse. Our results implicate that the walking pattern is not altered in patients with symptomatic external snapping hip. Hence, gait retraining may not be a reasonable treatment modality. Previously, we have demonstrated a significant strength deficit in hip abductors among patients with external snapping hip compared with healthy subjects [9]. It suggests that improvement of muscle strength and adequate balance of muscle function around the hip may be the first choice of treatment. Furthermore, the present study includes patient reported outcome measures, which provide important information for future studies in patients with external snapping hip.

### Limitations of the study

Even though the design of this study was based on commonly accepted methods some limitations need to be discussed. Several circumstances may mask a potential between-group difference. Subjects with external snapping hip all reported low pain during rest and activity indicating that the majority of subjects had mild symptoms at the time of examination. In addition, both groups walked at a self-selected speed, where differences can be hard to detect as kinetics and kinematics depend on walking speed [11,26]. Therefore it is possible that a higher walking speed or running could have resulted in larger differences between the groups especially because of the higher frontal forces during running [10,11]. No set of specific diagnostic criteria exist for the diagnosis of external snapping hip. Therefore we decided to include both subjective and objective inclusion criteria to minimize selection bias. Unfortunately this was only possible for the subjects with external snapping hip. The healthy subjects did not undergo radiographic or clinical examinations of the hip joint before inclusion, and although the risk is small it cannot be ruled out that some of the healthy subjects may have undiagnosed hip conditions. Finally, the sample size calculation was based on the peak hip adduction angle from a study on a different hip condition. Our data indicate that a larger sample size may be needed to detect between-group difference for the sEMG amplitudes. Therefore we cannot rule out that the observed trend of a reduced activity of the gluteus medius muscle exist due to chance alone. However, generally the between-group differences were small and may question if the small differences may be clinically meaningful.

## Conclusions

No significant differences were found in muscle activity around the hip or in kinetics and kinematics of the lower extremity between subjects with symptomatic external snapping hip and healthy subjects. This suggests that subjects with symptomatic external snapping hip do not show an impaired gait pattern.

## Competing interests

The authors declare that they have no competing interests.

## Authors’ contributions

All co-authors took part in the planning of this study, the test-period as well as the writing of this article. MU and MSR had special focus on the design and overall use of methods, while UGK had special focus on the biomechanical part of this study. KS and OS had special focus on the subjects with external snapping hip and process of diagnosis. As the first author JSJ was responsible for coordination of the different elements in this study and was responsible for the inclusion of the subjects and overall responsible for the decisions made in this study. All authors have read and approved the final manuscript.

## Pre-publication history

The pre-publication history for this paper can be accessed here:

http://www.biomedcentral.com/1471-2474/14/212/prepub
